# Instructions in the Preparation, Administration, and Properties of Nitrous Oxide, Protoxide of Nitrogen, or Laughing Gas

**Published:** 1866-10

**Authors:** 


Instructions in the Preparation, Administration, and Properties of Nitrous Oxide, Protoxide of Nitrogen, or Laughing Gas. By Geo. T. Barker, D.D.S., Prof. of Principles of Dental Surgery and Therapeutics, in the Pennsylvania College of Dental Surgery, etc., etc. Philadelphia: Rubencame & Stockton. 1866.

This is a monograph of 61 pages, printed on plain type and good paper. It is a very useful work for all such as desire familiar and reliable instruction in regard to the best methods of preparing and administering the Nitrous Oxide Gas.

